# Mapping the flow of veterinary antibiotics in Kenya

**DOI:** 10.3389/fvets.2024.1304318

**Published:** 2024-04-05

**Authors:** Alexina K. Morang’a, Dishon M. Muloi, Simon M. Kamau, Joshua O. Onono, Peter B. Gathura, Arshnee Moodley

**Affiliations:** ^1^Animal and Human Health Program, International Livestock Research Institute, Nairobi, Kenya; ^2^Department of Public Health, Pharmacology and Toxicology, Faculty of Veterinary Medicine, University of Nairobi, Nairobi, Kenya; ^3^Institute of Infection, Veterinary and Ecological Sciences, University of Liverpool, Liverpool, United Kingdom; ^4^Department of Veterinary and Animal Sciences, Faculty of Health and Medical Sciences, University of Copenhagen, Frederiksberg C, Denmark

**Keywords:** antimicrobial, Africa, distribution, governance, LMIC, policy, supply chain

## Abstract

**Introduction:**

To effectively regulate and reduce antibiotic use, in the livestock sector, a thorough understanding of the flow of veterinary antibiotics will help to identify key nodes in the chain for targeted interventions. The aim of this study was to understand the flow of antibiotics from import to end-user, and identify relevant governance mechanisms.

**Methods:**

A mixed methods approach was used to collect data in three Kenyan counties (Nairobi, Kiambu, and Kajiado). Focus group discussions (*n* = 23), individual interviews (*n* = 148), and key informant interviews (*n* = 10) were conducted.

**Results:**

The key actors identified include primary wholesalers, secondary wholesalers, retailers, animal health service providers (AHSPs), and farmers. Kenya imports 100% of its veterinary antibiotics: primary wholesalers legally import antibiotics as finished pharmaceutical products (90%) or active pharmaceutical ingredients (10%) after approval by the Veterinary Medicines Directorate. Secondary wholesalers play a major role in the distribution of antibiotics (60% of antibiotics) from importers to farmers, AHSPs, and retailers. Some of the illegal sources of antibiotics include unlicenced/unauthorized middlemen and online platforms that sell directly to retailers, AHSPs, and farmers.

**Discussion:**

Despite the presence of various laws and regulations governing the antibiotic value chain, implementation has been a challenge due to financial and human resource constraints. This contributes to over-the-counter sale of antibiotics without prescription, unlicensed businesses selling antibiotics, illegal importation, and presence of poor-quality drugs. There is a need to review the applicability of existing policies and address policy gaps (e.g., product containing antibiotic combinations, and use of human critically important antibiotics) to ensure the prudent sale and use of antibiotics, pharmacovigilance, antimicrobial use surveillance, and developing a business model that aligns with antibiotic stewardship. Additional interventions include awareness raising and capacity building of the different stakeholders along the antibiotic distribution chain to reduce antibiotic mis- and overuse.

## 1 Introduction

Antimicrobials are routinely used in livestock to maintain animal health, welfare, and productivity; however, antimicrobial misuse and overuse have been linked to the emergence and spread of antimicrobial resistance (AMR) (1). In 2019, it was estimated that 4.95 million deaths occured as a result of bacterial AMR, off which 22% occurred in Sub-Saharan Africa, however, the burden of AMR in animals is yet to be determined (2). The World Health Organization recognizes AMR as one of the top 10 global public health and development threats (3). Increased antimicrobial use (AMU) has been attributed to the increase in the number of animals for food production and the shift of farming practices from small-scale to intensive. Projections indicate that global AMU in food animals will increase by 67% by 2030 largely in Sub-Saharan Africa (SSA) (1). We are unable to quantify AMU in livestock in Kenya and the purpose of use as the country does not have a national AMU/C monitoring system. A few studies provide some indication on AMU at the retail and farm level, e.g., a study in Kiambu, Kenya, reported that 90% antibiotics were used for treatment of sick birds, and that tetracycline was the most used followed by tylosin, erythromycin, colistin, and neomycin (4). At the retail level in Nairobi, Kenya; tetracycline, sulfonamides, penicillin, and macrolides were commonly sold antibiotic classes (5). Despite several factors contributing to AMR, misuse and overuse of antimicrobials are the main driving factors (6). Demand for specific prescriptions, self-medication, absence or poor implementation of regulations, drug promotion by drug suppliers, irrational prescriptions, and faulty dispensing by the prescribers contribute to irrational AMU (7). Moreover, stocking of veterinary drugs on farm may result in poor storage and handling, thus reducing the efficacy of antimicrobials (8).

AMR has been recognized as a One Health concept, being intertwined into the agriculture, environment, and public health sector. Genomic studies conducted in Sub-Saharan Africa indicate that frequent unhygienic interaction between food animals and humans has resulted to the high transmission of AMR across livestock, the environment and human population (9).

The global recognition of AMR by the WHO as a public health threat resulted in the development of Global Action Plan (GAP) that sets out five strategic objectives and acts as a guide for the development of the national action plans (NAPs) on AMR (10). Kenya adopted its first AMR-specific policy in 2016 focused on improving AMR awareness and understanding in the community, strengthening AMR knowledge and evidence base through surveillance, decreasing infection incidences, optimizing antimicrobial use, and supporting research of new medicines, diagnostic tools, and vaccines. Reviewing and development of policies, guidelines, and legislation were among the proposed interventions for optimizing AMU (11).

Antimicrobial stewardship (AMS) is an important tool for mitigating AMR (12). AMS programs aim at optimizing antibiotic use, use of diagnostics to inform treatment decisions, and behavioral change regarding prescription and dispensing of antibiotics to limit the emergence, selection, and spread of AMR (10). Despite the implementation of antibiotic stewardship and surveillance programs in both human and animals, no significant progress has been achieved. Only 23 Africa countries, as per the WHO report have enrolled in the Global Antimicrobial Resistance and Use Surveillance System (GLASS) (3). Inadequate and inefficient data collection and surveillance systems in Sub-Saharan Africa has been attributed to the lack of comprehensive data on AMU and AMR (13, 14). Despite most countries strengthening regulations on antibiotic use and resistance as measures for improving AMS (15), implementation in most low- middle-income countries (LMICs) remains a challenge due to the limited enforcement capacity and diagnostic infrastructure (16).

Antimicrobial retailers and prescribers play a major role in AMU, and are on the frontline of AMS (17). In many high income countries, the pharmaceutical business is tightly regulated by the government to balance various objectives such as protecting the health of the population, enabling access to safe and effective medicine, and constraining pharmaceutical expenditure (18). In most LMICs, regulation and enforcement of the pharmaceutical business from registration, manufacture, sale and quality of medicines is weak. Despite efforts by the regulatory authorities to ensure guidelines for authorization, some perceive licenses as a source of revenue for the regulators and requires fewer resources than enforcing regulations that forbid over the counter sale of antibiotics without a prescription (19). AMU surveillance is critically important for effective, evidence-based policy, stewardship, and control of AMR, including monitoring the quality of veterinary medicines in the market. However, such surveillance and monitoring programs are still a challenge in most LMICs (20). To develop context-specific, effective strategies to reduce AMU, there is need to understand the flow of veterinary antibiotics, prescription/sale and access/use practices, and the regulatory mechanisms in place in a country. This study aims to determine the veterinary antibiotic flow, and understand the governance structure. Findings from this study will help key stakeholders, especially policymakers, to know more about the flow of antibiotics in the market, and hence, be able to develop effective and sustainable policies.

## 2 Materials and methods

### 2.1 Study area, design, and ethical approval

A cross-sectional study targeting stakeholders along the veterinary antibiotic distribution chain within Kenya with a focus on Nairobi, Kiambu, and Kajiado counties was conducted between November–December 2022. Nairobi is Kenya’s capital city and a key hub for drug manufacturers and regulatory agencies. Kiambu and Kajiado counties were selected based on their proximity to Nairobi and their differing farming systems. Kiambu County is characterized by intensive pig, poultry, and dairy production systems, while Kajiado has a more pastoralist production system.

### 2.2 Data collection

**Figure 1:**
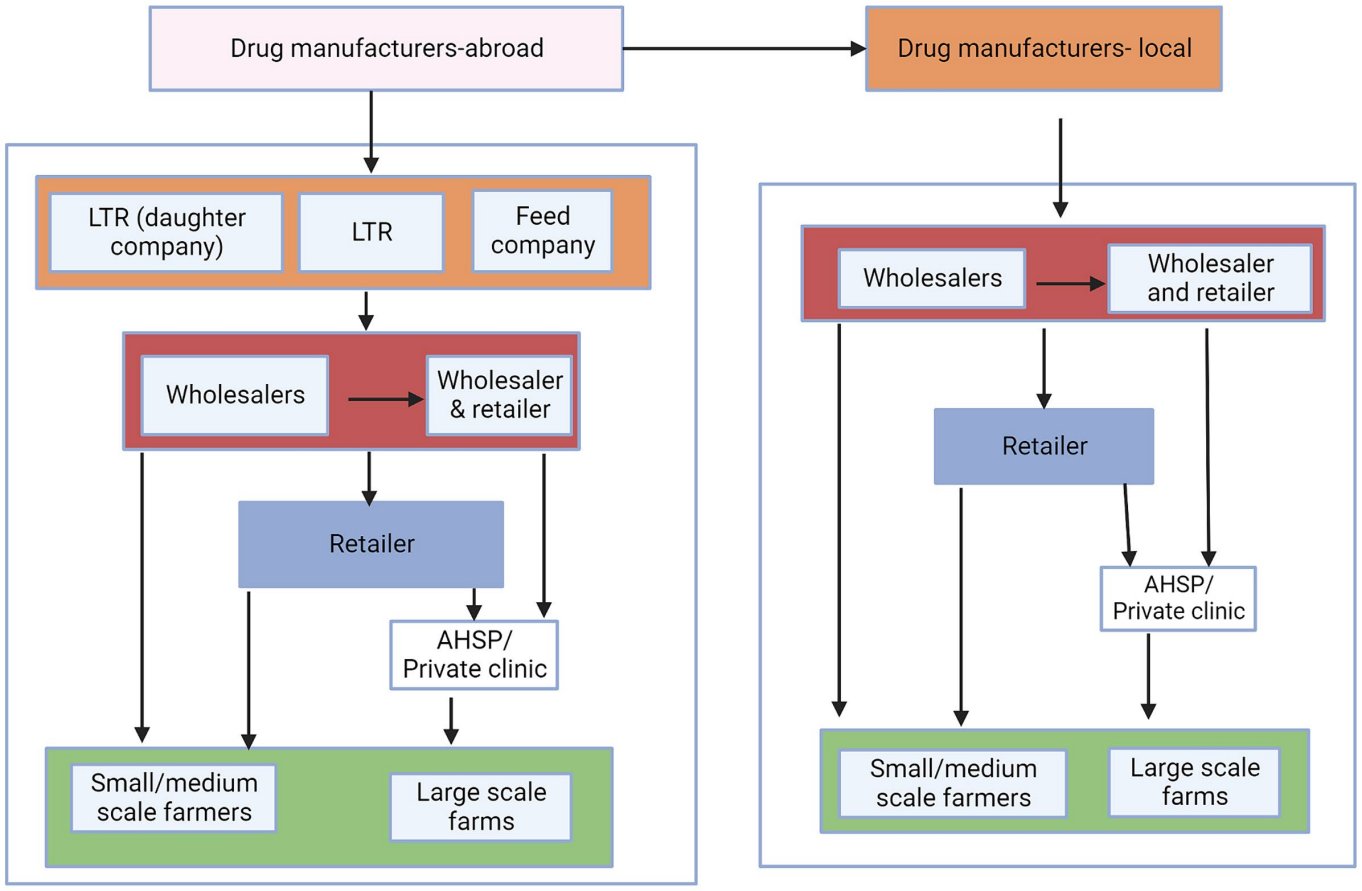
Conceptual map of the flow of veterinary antibiotics. LTR are local technical representatives, who assist foreign manufacturing companies in distributing their products in country. Animal feed companies represent feed manufacturers that incorporate antibiotics in the feeds while institutions represent research organizations, county government, and universities. The colors represent the different levels of the distribution chains.

A desk review was performed to provide an initial conceptual understanding of the possible flow of veterinary antibiotics and to identify relevant stakeholders along the value chain. This conceptual map, was also used during focus group discussions (FGDs) and key informant interviews (KIIs) to prompt questions for discussion purposes (Figure 1).

Primary data was collected by three methods: FGDs, individual interviews, and KIIs (Table 1).

**Table 1:** Summary of people interviewed during the study.

Type of interview	Category	Number of participants
FGDs and Individual interviews	Dairy farmers	25
	Pig farmers	23
	Poultry farmers	38
	Veterinary drug store workers/owners	22
	Animal Health Service Providers (AHSPs)	40
Key informant interview	Regulatory authorities	2
	Pharmaceutical companies	5
	Wholesalers	2
	Pharmaceutical association	1
	Large integrated poultry company	1

Focus group discussions were carried out with farmers, veterinary drug store workers/owners, and AHSPs to collect both qualitative and quantitative data. Participants were asked open-ended questions to describe their operations, map out the supply chain, estimate quantities of antibiotics, identify the roles and responsibilities of stakeholders and their interactions with other stakeholders, and discuss regulatory themes such as policies and regulations (Supplementary Table S1). Open-ended prompts such as “why” and “how” were utilized to encourage comprehensive discussions until a consensus was achieved. Additionally, individual interviews were carried out with the same FGD participants using a structured questionnaire to understand the personal perspectives about the veterinary antibiotic flow and the governance structures. To complement and validate FGD data, KIIs involving individuals from the regulatory authorities, pharmaceutical companies, wholesalers, pharmaceutical associations, and large-scale farms were conducted. KII participants were deemed stakeholders with first-hand knowledge about the veterinary antibiotic supply chain and the governance structures. A similar approach as in the FGDs, was used for the KIIs, but with additional questions asking participants to describe their roles and their potential influence on the antibiotic supply chain. All qualitative data from FGDs and KIIs were captured through audio recordings and informed consent from the participants was obtained.

### 2.3 Data management and analysis

Audio recordings were carefully listened to and data in notebooks and flip charts read before being collated into a Word document around key themes related to the study objectives, and transcribed into preformatted templates organized around: (1) disease management and source of antibiotics, (2) patterns of antibiotic storage, handling and disposal, (3) recording keeping on antibiotic sale, (4) stakeholder interactions, and (5) knowledge on the regulatory bodies and existing regulations. Based on the data from the templates, we (a) refine the veterinary antibiotic flowchart and (b) identify critical gaps including level of regulatory implementation, stakeholder interaction, practices and knowledge gaps. A detailed antibiotic flow chart was generated by compiling information from the templates. The antibiotic flow chart was created using RawGraphs and Adobe Illustrator software. Different laws and regulations on antimicrobials were identified and the level of implementation was assessed based on participants’ knowledge, practice, and insight of the regulatory authorities. They also provided a better understanding of the roles of the regulatory authorities and the chain of command. The rules governing the veterinary pharmaceutical business were classified into two distinct types: formal regulations encompassing Acts and Regulations, and informal regulations comprising company policies. We defined an Act as a law that clearly defines the rules of individual behavior, the powers and limitations of public bodies, and the rights of individuals subject to those powers. Similarly, regulations were defined as secondary legislations that have less authority and provide details on how an Act will be implemented. Lastly, policies were defined as statements of intent that identify institutional goals and the appropriate mechanisms for achieving them, but they do not have the force of law (21). Data from the individual questionnaires were entered into Microsoft Excel for cleaning and analysed using descriptive statistics. The interactions among stakeholders were visualized using Social Network Visualizer v3.1 software.

### 2.4 Data validation

Initial results from the FGDs were presented during KIIs to better understand some of the gaps that were identified. Findings from this study were also compared to similar studies in other countries such as Uganda and Lao, however it was noted that most of the findings were similar, but there were some slight differences. Results were also shared to other researchers to critic and check for biasness.

## 3 Results

### 3.1 Structure and flow of veterinary antibiotics

The veterinary antibiotic value chain includes public and private sectors actors. Public sector actors comprises of the regulatory authorities, who are responsible for developing laws and enforcement, overall regulation of the veterinary pharmaceutical business in Kenya, guidelines for antimicrobial stewardship practices, and conducting AMU and AMR surveillance in animal health. They include the Veterinary Medicine Directorate (VMD), Kenya Veterinary Board (KVB), and Directorate of Veterinary Services (DVS). Private sector actors include primary wholesalers, secondary wholesalers, retailers, middlemen, human pharmacies, online platforms, and end-users and are governed by a set of laws (namely the VSVP Act 2011), with VMD responsible for enforcement. Primary wholesalers are defined as large-scale pharmaceutical businesses located in country; aiding drug manufacturers primarily located abroad to locally distribute their products. Secondary wholesalers are medium-scale pharmaceutical businesses that mainly re-distribute drugs to retailers who sell in smaller quantities to the end-users (farmers, AHSPs). Middlemen and online platforms, which are illegal vendors that sell antibiotics directly to farmers, AHSPs, and retailers.

**Figure 2:**
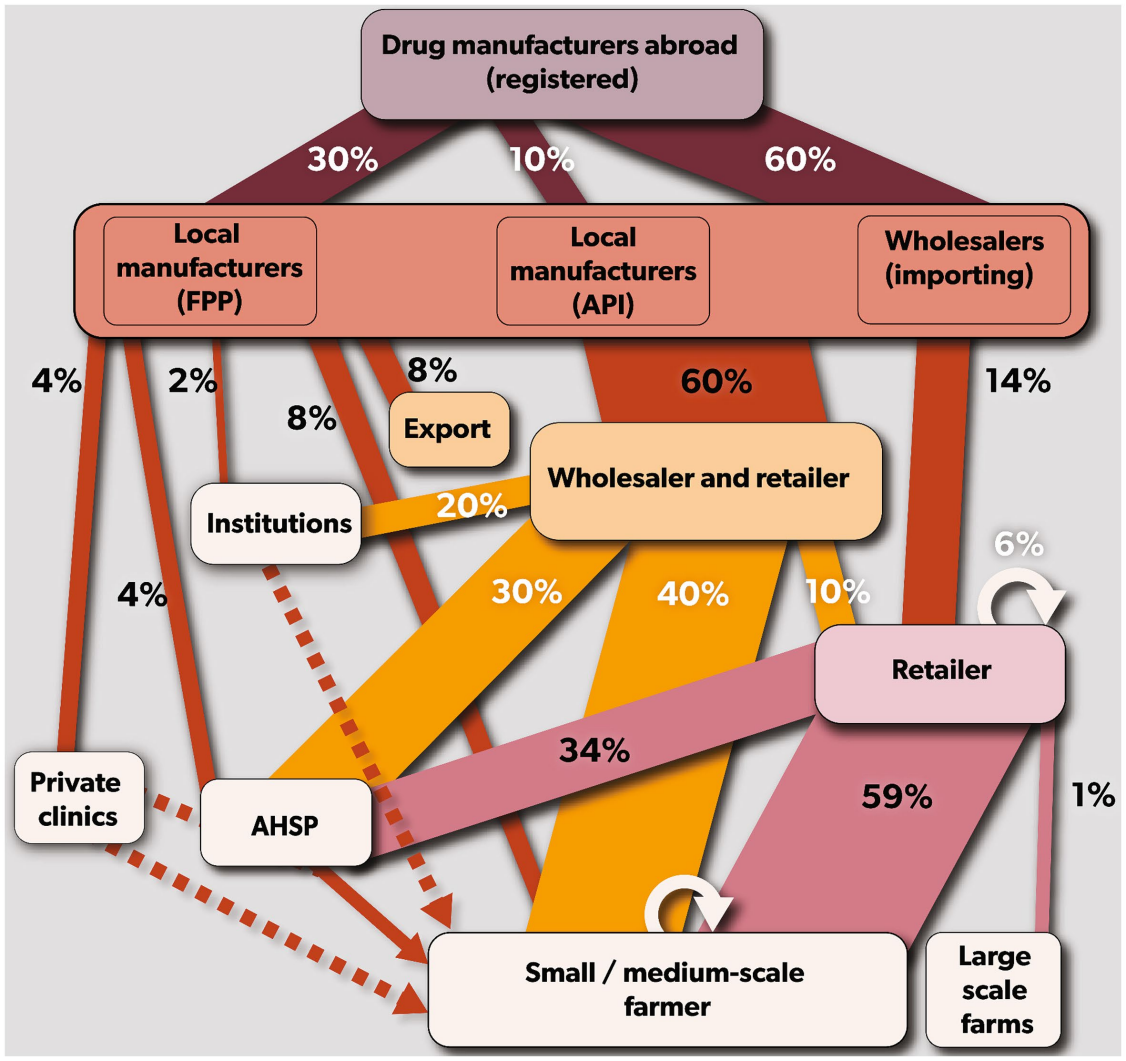
Mapping of veterinary antibiotics supply chain in Kenya. Rectangular boxes represent the stakeholders (drug manufacturer = violet, primary wholesalers = coral red, secondary wholesaler = yellow and retailers = purple). The arrows (drug manufacturers = maroon, primary wholesalers = brown, secondary wholesalers = orange, and retailers = purple) indicate the antibiotic flow from each stakeholder. The size of the arrows indicates the average percentage of antibiotics in that chain.

Kenya relies on the importation of veterinary antibiotics as it does not produce any antibiotics domestically. These antibiotics are sourced from international producers and are either imported as finished pharmaceutical products (FPPs) or active pharmaceutical ingredients (APIs). Of all the antibiotics imported, 60% are in the form of FPPs and are ready to be sold, while 30% are imported as FPP but are repackaged or relabelled before domestic sale, and lastly, the remaining 10% are imported in the form of APIs that are processed into FFPs before sale. Once in the country, 60% of both FFPs and APIs, are primarily distributed through secondary wholesalers, who sell them in bulk to retailers (e.g., veterinary drug stores) or directly to farmers and AHSPs. The remaining 40% is distributed either directly to retailers (14%), farmers (8%), AHSPs (4%), private veterinary clinics (4%), institutions (2%) or exported to regional markets (8%). Across the supply chain, representatives of the primary wholesalers who are locally known as ‘sales reps’ facilitated the movement of antibiotics through the value chain (Figure 2).

**Figure 3:**
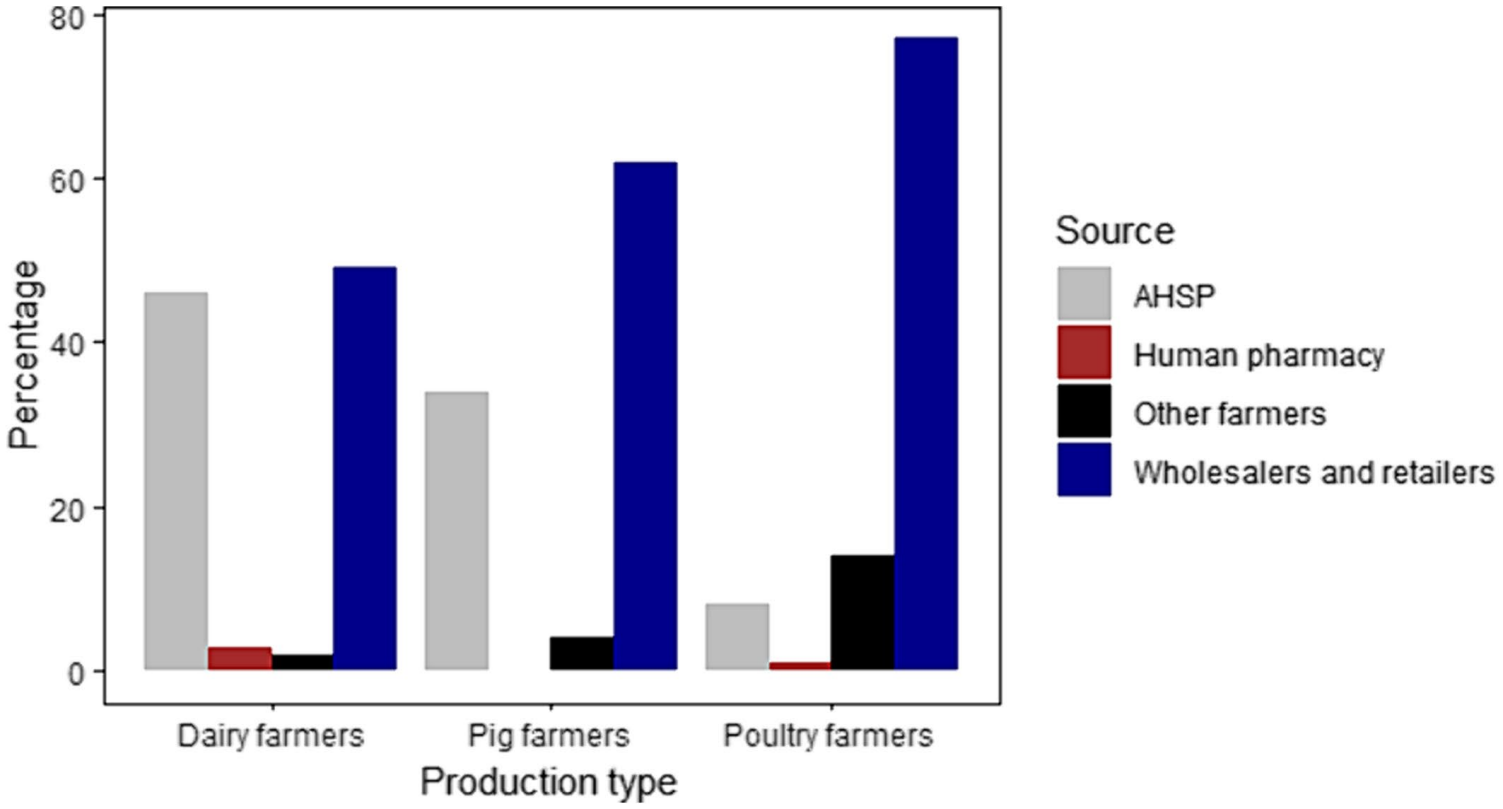
Graphical representation of the different sources of antibiotics for dairy, pig, and poultry farmers.

Many farmers reported buying their antibiotics mainly from retailers (Figure 3). Poultry farmers also reported sharing (14%) antibiotics with each other. Similarly, pig farmers in Ruiru (10%) and Ngong (10%), and Ngong dairy farmers (10%) reported sharing antibiotics. The use of human designated drugs was reported by dairy farmers at Gathundu South (10%) and Ngong (5%), and poultry farmers in Ngong (5%), however, all pig farmers reported not administering “human” medicines to their animals. A large-integrated poultry company, with flock sizes >30,000 birds, reported sourcing antibiotics from retailers and secondary wholesalers. These antibiotics were only used by company veterinarians on contract farms and on birds after examination and diagnosis.

### 3.2 Governance

#### 3.2.1 Formal regulation and policies

The veterinary pharmaceutical industry in Kenya is regulated by VMD, as stipulated by the Veterinary Surgeon and Veterinary Paraprofessional (VSVP) Act of 2011. This includes all activities along the antibiotic supply chain, from importation to distribution, inspection and approval of veterinary pharmacy premises, registration of veterinary drugs, and pharmaco-vigilance. Conversely, DVS and KVB regulate AMU practices in animals and responsible for ensuring adequate training of veterinary professionals in management of veterinary medicines, respectively. DVS and KVB are also members of the VMD council, and all institutions individually report to the Cabinet Secretary for Livestock.

According to the 2015 VMD Regulations, a person can apply for a veterinary pharmacy license only if he/she is a veterinary surgeon (i.e., holds a degree in veterinary medicine) or an equivalent qualification as determined by VMD council. A veterinary pharmacy, which includes manufacturers, wholesalers, and retailers, is a business authorized by VMD to stock, dispense and/or distribute veterinary medicines. The veterinary pharmacy business can only be owned/licensed by a veterinary surgeon or a veterinary paraprofessional. To trade veterinary medicines in bulk, a person must provide evidence that the business is under the management of a veterinary surgeon who is authorized to own a veterinary pharmacy business. A veterinary pharmacy with a wholesalers’ permit may sell antibiotics in bulk to registered retail dealers who may then go on to sell to the public on a prescription basis. Antibiotics are classified by VMD into Category I and Category II depending on the pharmaceutical formulation, route of administration, and who is allowed to prescribe them, e.g., injectable antibiotics are in Category I and may only be prescribed by a veterinary surgeon. However all antibiotics irrespective of the formulation or route of administration should only be sold with a prescription from a veterinary surgeon or person with equivalent qualification as determined by VMD.

#### 3.2.2 Implementation of laws and regulations and challenges encountered

To import or export antibiotics, including distribution and sales is governed by VMD. Some private sector stakeholders highlighted deficiencies in the enforcement of VMD regulations, with several respondents offering anecdotal evidence of illegal importation, distribution, and sale of antibiotics. Most pharmaceutical companies reported that they prefer importing antibiotics in the form of FPP rather than API due to the high cost of domestic production and the lengthy registration period. It was also noted that representatives of pharmaceutical companies, i.e., the primary wholesale companies employ ‘sales reps’ to assist in moving their antibiotic products along the value chain. This practice was viewed as a marketing strategy for the pharmaceutical business model and is not regulated. Furthermore, retailers and end users reported that antibiotics were often sold without prescriptions, which is against the regulations. Veterinary drug store owners reported that antibiotic sales were integral for their business profits and that certain pharmaceutical companies provided incentives for surpassing sales targets. Some (13.6%, 3/22) veterinary drugs stores reported lack of compliance with VMD business registration requirements and in (18.2%, 4/22) of the stores, the retail staff lacked animal health training. Farmers, veterinary drug store owners, and AHSPs expressed concerns about the quality of available antibiotics in the country, noting discrepancies in color, price, and labeling formats for the same brand and when there are cases of treatment failure. The VMD reported the possible presence of poor-quality drugs on the market to illegal importation across the porous borders and constraints to implement proper pharmacovigilance, for example few labs in Kenya are able to conduct drug quality testing and the approximate cost per analysis is $380. Lastly, most (68.2%, 15/22%) veterinary drug stores and (60%, 24/40) AHSPs reported a lack of awareness regarding antibiotic regulations and guidelines, as well as the lack of formal antibiotic usage and treatment guidelines.

During our KIIs, we identified a few policy gaps (i) combinations of multivitamins with antibiotics, e.g., Product A which contains oxytetracycline and is marketed to boost vitality and prevent chick mortality (Supplementary Table S2); (ii) combinations of >1 antibiotic, e.g., Product B (Supplementary Table S3); (iii) the use of human critically important antibiotics (CIAs) such as colistin in animals for prophylactic purposes; and (iv) marketing and labeling of antibiotic and multivitamin combinations. VMD reported that they are in the process of drafting a new policy to regulate antibiotic sales including registration and sale of multivitamins containing antibiotics.

### 3.3 Stakeholder interaction

**Figure 4:**
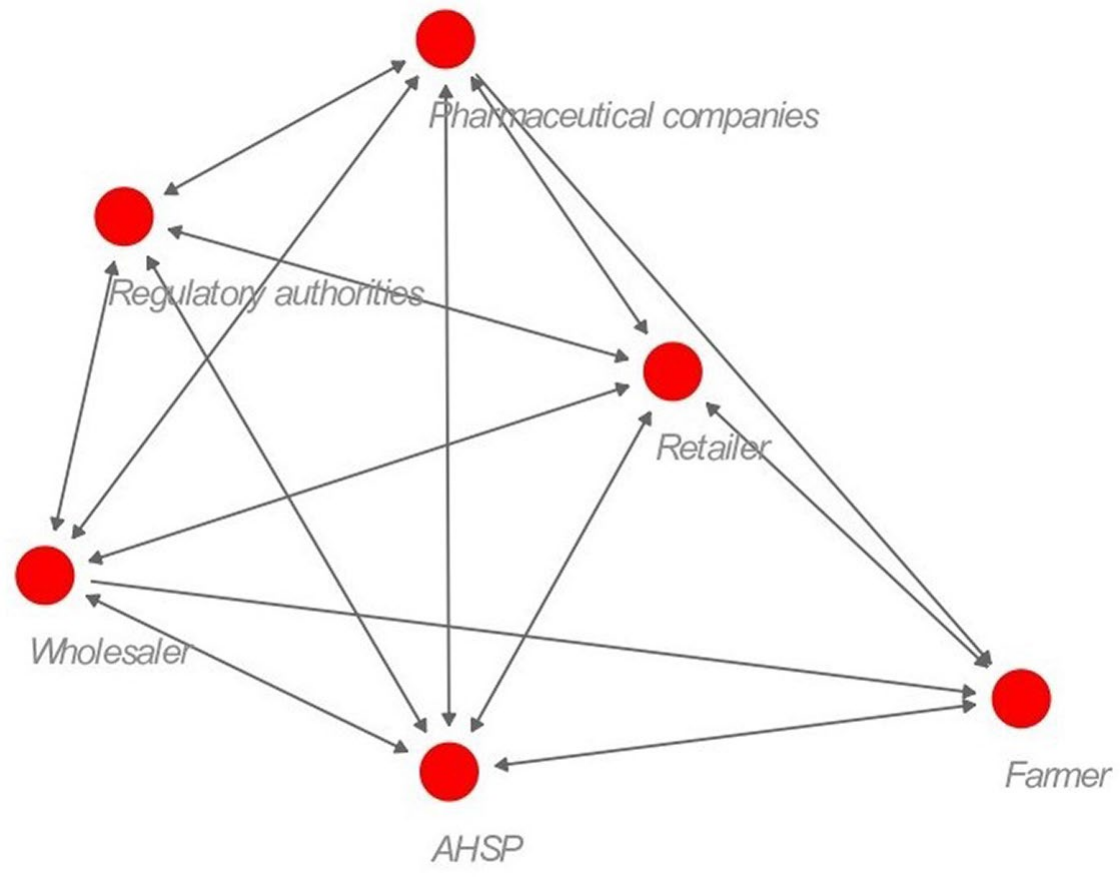
Social network structure for the veterinary antibiotic supply chain actors. The distance between the nodes denotes the closeness of interaction between the stakeholders. A double-ended arrow indicates both stakeholders mentioned interacting with each other. The red circle represents the different stakeholders.

AHSPs and veterinary drug stores (retailers) were the key stakeholders that interacted with all the other stakeholders (Figure 4). Interaction between farmers and regulatory authorities was minimal, which is expected, and most farmers were not aware of who regulates veterinary drugs in Kenya. Wholesalers and retailers reported having interacted with VMD only once a year during inspection and licensing, and most trainings and workshops they attended were organized by pharmaceutical companies as a company marketing strategy.

### 3.4 Practice and knowledge gap

#### 3.4.1 Sale and purchase

Retailers and wholesalers reported that the types of antibiotics sold were influenced by quality, price, demand, availability, and recommended storage method while antibiotic importation was influenced by market demand and seasonal variation. Some (9/22, 40.9%) veterinary drug stores reported that they sold antibiotics to farmers in portions instead of whole packages, depending on their demand. Most farmers (50/70, 71.4%) who used vitamins were unaware that some formulations also contained antibiotics. However, most of the veterinary drug store workers (20/22, 90.9%) were aware of this but still sold the products to farmers as well as additional antibiotic treatments. Most poultry farmers reported that they were advised by veterinary drug stores (purveyors of day-old chicks) and some hatcheries to use antibiotics for prophylactic purposes within the first 5 days to prevent disease and/or chick death.

#### 3.4.2 Knowledge on drug storage and disposal

Most stakeholders had low knowledge of proper storage and disposal of expired drugs. The commonly reported methods of disposal were throwing antibiotics into a pit latrine, burning them, throwing them in the dustbin, returning them to the distributor, or pouring them down the drain. A small proportion (4.5%, 1/22) of veterinary drug stores sold expired drugs at a discounted price while AHSPs and farmers reported that they continued to use drugs beyond their expiration date.

#### 3.4.3 Disease diagnosis and treatment

Poultry farmers reported that they frequently self-diagnosed and treated their sick birds with antibiotics, mimicking the practices of other farmers, and only relying on AHSPs when their treatments were ineffective. Despite the frequent practice of self- diagnosis, nearly all farmers did not know the name of the antibiotics they were using and instead described them by their package appearance or carried an empty sachet from a previous treatment or prescription note when they last purchased antibiotics. Diagnostic services were rarely utilized among the surveyed AHSPs, veterinary drug store workers and farmers, often choosing to practice empiric treatment based on observed symptoms. The barriers to accessing diagnostic services was attributed to cost, logistics as laboratories are located far from the farms, and lengthy test turnaround times. Farmers reported that they did not observe the recommended withdrawal period and some farmers acknowledged selling products such as milk and eggs that were likely contaminated with antibiotic residues. AHSPs reported rarely completing the recommended treatment schedule nor conduct case follow-ups, while most farmers (52/86) stopped treatment once the animal showed good clinical improvement.

## 4 Discussion

The private sector plays a pivotal role in the importation and distribution of antibiotics. Kenya imports 100% of all antibiotics mainly in the form of FPPs. The high cost of production and the lengthy drug registration period were the main reasons why most of the pharmaceutical companies preferred importing antibiotics in the form of FPPs. Most LMICs also prefer importing antibiotics rather than producing them locally due to stringent regulations, high cost of production, and narrow profit margins (19). Middlemen, who are illegal traders, play an important role in supplying antibiotics to veterinary drug stores, AHSPs, and farmers. Similarly, in Lao, Poupaud (22) showed that middlemen interacted with key stakeholders along the supply chain and were not regulated, reflecting how the veterinary drug business is operated by individuals who may not have the required qualifications and limited knowledge of antibiotic stewardship. There is need to enforce the laws and regulations to ensure that antibiotics are sold by qualified persons to ensure proper handling and prescription, and storage of drugs.

Kenya has successfully developed laws and regulations governing the veterinary pharmaceutical business. Despite the presence of these laws and regulations, we identified a few policy gaps: (i) sale of combinations of multivitamins and antibiotics, (ii) combination containing of >1 antibiotic class, (iii) use of human CIAs in animals and (iv) questionable marketing and labeling of combinations of antibiotic and multivitamins. Mutua (23) reported that the sale of antibiotic formulations containing >1 antibiotic was a common practice, e.g., Aliseryl™ which contains four antibiotics, namely erythromycin (macrolide), streptomycin (aminoglycoside), oxytetracycline (tetracycline) and colistin (polymyxin B). Similarly, Kariuki (24), identified a significant proportion of antibiotic brands (53.3%) on the Kenyan market for poultry use contained >1 antibiotic class. Antibiotic combinations has been attributed to the emergence of multiple drug-resistant bacteria due to exposure of bacteria to different antibiotic classes (25). Colistin, a CIA used as a last resort for treating multi-drug resistant bacterial infections, combined with other antibiotics, and used for prophylactic purposes in animals is of concern. Veterinary use of colistin has been banned in China, Bangladesh and Pakistan (26) due to concerns about mobile resistance genes (e.g., *mcr-1*) conferring colistin resistance in *Escherichia coli* of animal origin (27). To reduce veterinary use of human CIAs, Denmark banned the use of fluoroquinolones and an industry-led voluntary ban on the use of 3rd and 4th generation cephalosporins in pig production. To reduce animal AMU, a combination of mandatory and voluntary actions (bans and restricts on antibiotic use), strong antimicrobial stewardship programs and a focus on farm biosecurity and herd health have been shown to be successful, e.g., a 50% reduction of AMU in livestock between 2014 and 2021 in the United Kingdom (28). Non-compliance practices such as sale of antibiotics without a prescription, unlicensed veterinary pharmacies and unqualified professionals selling drugs were reported despite the presence of laws and regulations prohibiting this. Most LMICs have policies/laws that govern various aspects of the antibiotic supply chain but lack the resources to effectively enforce those policies, impose penalties on the defaulters, verify the quality of drugs entering in the country and identify counterfeit drugs (29).

The veterinary drug business is a profit-oriented business. Most veterinary drug stores sell antibiotics without a prescription for fear of losing customers and business, and pharmaceutical companies giving incentives to their distributors to increase sales and employ sales representatives who assist in marketing their products to generate more sales. Employment of sales representatives by pharmaceutical companies to market their products has been viewed as a norm and is not regulated in Kenya. A study from Vietnam showed that there were more drug prescriptions by private healthcare providers than by public health providers due to commercial and profit driven interests (30). Additional research is required to assess how financial incentives and different antibiotic profit margins can influence the decision on antibiotic prescription by AHSPs and drug retailers in LMICs. Also, more research is required to understand how business models could align with antibiotic stewardship, e.g., a model focused on delivering animal health services, including a focus on disease prevention measures rather than profit from antibiotic sales.

In most LMICs, counterfeit drugs are a major challenge (31, 32). Changes in the drug appearance, package labeling, sold at a cheaper price, and treatment failure are some of the indicators used by the stakeholders to suspect counterfeit drugs. Frost et al. (19) reported that poor quality antibiotics in less regulated markets are attributed to poor manufacturing practices of the pharmaceutical companies. Use of counterfeit products compromises public health (33) and contributes to AMR and drug resistant infections (34). Weak post-market surveillance due to financial constraints and insufficient laboratory capacity has resulted in difficulty in assessing the quality of drugs on the market. In this regard, regulatory bodies and pharmaceutical companies should work in harmony and conduct frequent pharmacovigilance to ensure on good quality products on the market.

Awareness of proper antibiotic handling, storage, and disposal is important to ensure the quality of the active compounds and hence the drug efficacy is maintained and minimizing treatment failure, development of resistance and reduce environmental contamination. Most stakeholders interviewed had limited knowledge on proper storage and disposal of drugs and how inappropriate storage alters the effectiveness of the drug. Poor storage of antimicrobials has been shown to increase the risk of degradation and increased chances of underdosing (35), production of toxic degradation compounds and delayed recovery resulting in increased morbidity and mortality (36). Non-professionals handling drugs, poor communication of the policies to the stakeholders, and inadequate consultation during policy development are some of the contributing factors to limited stakeholder knowledge.

Similar to the observations by Carron (37) in the broiler meat system in Nairobi, Kenya, drug retailers were frequently the first point of contact for farmers both for assistance with diagnosis and antibiotic purchase. Most of the farmers we interviewed preferred purchasing drugs directly from veterinary drug stores, often without prescription, and self-treat rather than seeking animal health advice from an AHSP. Similarly, in the human health sector, pharmacies have emerged as the primary point for outpatient healthcare offering unauthorized consultations, diagnosis, prescription, and dispensing of medications (38). The close relationship between farmers and retailers is important in the flow of antibiotics hence the need for specific consideration of this interaction during the development of interventions toward reducing AMU. Focusing on the enforcement of policies/regulations is not enough to regulate antibiotic access and use, and often key stakeholders are not consulted or excluded. A study from Lao in the health sector, showed that to increase the probability of successful and sustainable health policy reforms, diverse stakeholder groups should be involved in the policy design and implementation (39). Public-private partnerships and collaborative efforts are also important in monitoring informal channels. To develop effective antimicrobial stewardship programs, there is need for the involvement of all stakeholders along the supply chain to ensure inclusion of knowledge, experience and available resources and capacities.

There are limitations to our study. Firstly, the selection of participants for the FGDs and individual interviews were done by the government veterinary officers and was based on the likely existing relationship with the participants and having previous contact in other research activities. This could result in biases. Secondly, only two wholesalers and one large integrated poultry company participated in the KIIs thus limiting the variation in information captured. However, during the interviews we realized that most of the wholesalers also operate as retailers, hence the similarity in data collected at the retail level.

## 5 Conclusion

Here, we describe how veterinary antibiotics are distributed across the various channels and the stakeholders involved along the supply chain in Kenya. The veterinary pharmaceutical business plays a critical role in the livestock sector through the provision of medicines for treatment, prevention, and growth promotion. Antibiotic sales are tightly linked to retailers and AHSPs profit margins and for farmers, use antibiotics are linked to a protection of livelihoods (40). Despite, the existence of laws and regulations governing antibiotic access and use in Kenya, implementation is a challenge. This contributes to illegal veterinary business practices such as over-the-counter sales of antibiotics without a prescription, proliferation of counterfeit and sub-standard drugs on the market, illegal businesses, and illegal importations. Frequent stakeholder sensitization on AMS, development of a business model that aligns with antimicrobial stewardship, development of treatment guidelines, surveillance of antimicrobial consumption, involvement of stakeholders during policy development and behavioral change that focus on improving animal health and farm management are some of the efforts to be put in place toward optimizing AMU. Findings from this study provide suggestions to policymakers on the potential areas of interventions to ensure better regulation of the veterinary drug business. In addition, all stakeholders should be involved during policy development for inclusivity and ease of implementation process.

## Data availability statement

The raw data supporting the conclusions of this article will be made available by the authors, without undue reservation.

## Ethics statement

The study was conducted in accordance with local legislation and institutional requirements. Studies involving humans were approved by the International Livestock Research Institute—Institutional Research Ethics Committee (ILRI- IREC) with project reference project reference: ILRI-IREC2022-33. ILRI-IREC is accredited by the National Commission for Science, Technology and Innovation (NACOSTI) in Kenya. In addition, a research permit was obtained from NACOSTI (License No: NACOSTI/P/22/19437). Written informed consent for participation in this study was provided by the participants.

## Author contributions

AKM: Writing – original draft, Writing – review & editing. DMM: Supervision, Writing – review & editing. SMK: Writing – review & editing. JOO: Supervision, Writing – review & editing. PBG: Supervision, Writing – review & editing. ArM: Funding acquisition, Supervision, Writing – review & editing.
