# Supplementary material for: Mapping the flow of veterinary antibiotics in Kenya
Source: Front Vet Sci. 2024 Apr 5;11:1304318. doi: 10.3389/fvets.2024.1304318 (PMC11027570; doi:10.3389/fvets.2024.1304318)
Supplement: Supplementary file 1 [file Data_Sheet_1.PDF]

Supplementary Tables

Supplementary Table S1

1. FGDs guide for farmers

Participants

Person ID	Description
1	
2	
3	

Demographics

1. Which farming systems (poultry, beef, dairy, pig) do you engage in?
2. How many years in production?
3. Training in animal production/motivation for farming

Animal health/Antibiotic use

4. What do you do when you encounter diseases within your farm?
5. How often do you use/buy antibiotics?
6. Which antibiotics do you commonly use?
7. What are the reasons for using antibiotics and what informs the choice?
8. Where do buy the antibiotics from/reasons for this source?
9. Is this source reliable?
10. How do you store your antibiotics?
11. What do you do when antibiotics fail to work?
12. Do you complete the recommended dosage for antibiotics?
13. What challenges do you face in accessing antibiotics?

Access to animal health services

14. Where do you get advice regarding the use of antibiotics?
15. What kind of advice are you given regarding antibiotic use?
16. How often do you call your vet/animal health practitioner?
17. How often do you encounter counterfeit antibiotics?
18. What do you think contributes to the presence of counterfeit antibiotics?
19. How often do you attend training?
20. Who organizes such training?

Governances and interactions

21. What is your take on the effectiveness of the existing policies on antibiotic use?
22. Which other stakeholders do you interact with, and what type of interaction?

Stakeholder category	Description
1 Farmer	
2 Trader	
3 Vet /animal health practitioner	

4. Agrovet owner/worker	
5.	

2. **FGDs guide for veterinary drug store workers/owners**

Participants

**Person ID**	**Description**
**1**	
**2**	
**3**	

Demographics

How many years in business?

How often do you attend training and how have they benefited you?

Animal health/Antibiotic sale

1. Which antibiotics do you commonly sell?
2. Where do source antibiotics from?
3. What informs the choice of antibiotics to sell?
4. What dictates the price of antibiotics?
5. How do you store your antibiotics?
6. What are the common practices regarding antibiotic use?
7. Which challenges do you encounter in accessing antibiotics?
8. Who are your main customers?

Access to animal health services

9. What kind of advice do you give to clients/farmers regarding antibiotic use?
10. How often do you encounter counterfeit antibiotics?
11. What do you think contributes to the presence of counterfeit antibiotics?
12. What are the benefits of record keeping on antibiotic sale/use?
13. Which other stakeholders do you interact with and what type of interaction?

Stakeholder category	Description
1 Farmer	
2 Trader	
3 Vet /animal health practitioner	
4. Agrovet owner/worker	
5.	

Governance structure and interactions

14. Who oversees the regulation of antibiotic use?
15. How often do these bodies conduct monitoring and evaluation and what is the impact?
16. What are some of the benefits of having policies on antibiotic use?
17. What is your take on the effectiveness of the existing policies on antibiotic use?
18. What challenges affect the functionality of the existing policies?
19. What do you think should be done to improve the interaction between stakeholders and the relevant regulating bodies?

3. FGDs guide for AHSPs

Participants

**Person ID**	**Description**
**1**	
**2**	
**3**	

Demographics

How many years in business?

How often do you attend training and how have they benefited you?

Animal health/Antibiotic use

1. Which antibiotics do you commonly sell/use?
2. Where do you source the antibiotics that you sell/use?
3. What informs the choice of antibiotics that you sell/use?
4. What dictates the price of antibiotics that you sell/use?
5. Which challenges do you encounter in accessing antibiotics?
6. Who are your main customers?
7. What are the common practices regarding antibiotic use?

Animal health services

8. What kind of advice do you give to clients/farmers regarding antibiotic use?
9. What are the benefits of proper record keeping on antibiotic sale/use?
10. How often do you encounter counterfeit antibiotics?
11. What do you think contributes to the presence of counterfeit antibiotics?
12. How often do you attend training?
13. How has this training benefited you?
14. Who organizes such training?
15. Which stakeholders do you interact with and what type of interaction?

Stakeholder category	Description
1 Farmer	
2 Trader	
3 Vet /animal health practitioner	

4. Agrovet owner/worker	
5.	

16. What are your roles along the antibiotic supply chain?

Governance structure and interactions

17. Who oversees the regulation of antibiotic use?
18. How often do these bodies conduct monitoring and evaluation and what is the impact?
19. What are some of the benefits of having policies on antibiotic use?
20. What is your take on the effectiveness of the existing policies on antibiotic use?
21. What challenges affect the functionality of the existing policies?
22. What is your take on the private-public partnership pertaining to antibiotic policy implementation?
23. What do you think should be done to improve the interaction between stakeholders and the relevant regulating bodies?
